# Supplementary material for: Cross-Reactive Fc-Mediated Antibody Responses to Influenza HA Stem Region in Human Sera Following Seasonal Vaccination
Source: Vaccines (Basel). 2025 Jan 28;13(2):140. doi: 10.3390/vaccines13020140 (PMC11860798; doi:10.3390/vaccines13020140)
Supplement: Supplementary file 1 [file vaccines-13-00140-s001.zip › vaccines-3421382-supplementary.pdf]

Cross-reactive Fc-mediated Antibody Responses to Influenza HA Stem Region in Human Sera Following Seasonal Vaccination

Supplementary Table S1. Characteristics of study participants.

Characteristics of study participants.	All (N=50)
	n (%)
**Sex**	
Male	10 (20%)
Female	40 (80%)
**Age(years)**	
Median (range)	47.5 (64-22)
20-29	3 (6%)
30-39	5 (10%)
40-49	21 (42%)
50-59	16 (32%)
60-69	5 (10%)
**BMI (kg/m^2)**	
Median (range)	21.0 (30.1-16.6)
Underweight (<18.5)	8 (16%)
Normal (18.5-24.9)	35 (70%)
Overweight (≧ 25)	7 (14%)

Supplementary Table S2. H1 LAH and H3 LAH peptide sequences from H1N1 (A/Guangdong-Maonan SWL1536/2019) and H3N2 (A/Hong Kong/2671/2019).

Virus strain	Amino acid sequence
H1N1 A/Guangdong-Maonan SWL1536/2019 LAH ;	RIENLNKKVDDGFLDIWTYNAELLVLLENERTLDYHDSN VKNLYEKVRNQLKNNA
H3N2 A/Hong Kong/2671/2019 LAH ;	RVQDLEKYVEDTKIDLWSYNAELLVALENQHTIDLTDSE MNKLFEKTKKQLRENA

ID	Group1 score						Group2 score						G1+2
	Vic	GM	Cal04	PR8	H5	**Total**	Dar	HK	GZ	X31	H7	**Total**	**Total**
1	1	1	1	0	0	**3**	2	2	4	0	0	**8**	**11**
2	1	1	1	0	0	**3**	1	1	2	0	0	**4**	**7**
3	2	1	1	0	0	**4**	1	1	2	0	0	**4**	**8**
4	2	1	1	0	0	**4**	1	2	1	0	0	**4**	**8**
5	1	2	1	0	0	**4**	1	2	1	0	0	**4**	**8**
6	1	0	1	0	0	**2**	1	2	0	0	0	**3**	**5**
7	1	1	2	0	0	**4**	1	2	2	0	0	**5**	**9**
8	2	2	1	0	0	**5**	2	2	2	0	0	**6**	**11**
9	2	1	1	0	0	**4**	2	1	2	0	0	**5**	**9**
10	1	1	0	0	0	**2**	2	2	3	0	0	**7**	**9**
11	1	1	1	0	0	**3**	1	1	1	0	0	**3**	**6**
12	1	2	1	0	0	**4**	2	2	1	0	0	**5**	**9**
13	2	2	1	0	0	**5**	1	1	2	0	0	**4**	**9**
14	1	1	1	0	0	**3**	2	1	2	0	0	**5**	**8**
15	1	2	1	0	0	**4**	1	4	2	0	0	**7**	**11**
16	1	0	1	0	0	**2**	2	2	1	0	0	**5**	**7**
17	2	0	1	0	0	**3**	2	4	1	0	0	**7**	**10**
18	1	0	1	0	0	**2**	1	2	2	0	0	**5**	**7**
19	1	1	1	0	0	**3**	1	1	2	0	0	**4**	**7**
20	1	0	1	0	0	**2**	2	2	2	0	0	**6**	**8**
21	1	0	1	0	0	**2**	1	2	2	0	0	**5**	**7**
22	2	0	1	0	0	**3**	2	1	3	0	0	**6**	**9**
23	1	0	1	0	0	**2**	1	2	0	0	0	**3**	**5**
24	1	0	1	0	0	**2**	1	0	2	0	0	**3**	**5**
25	1	0	1	0	0	**2**	1	1	2	0	0	**4**	**6**
26	2	0	1	0	0	**3**	1	3	2	0	0	**6**	**9**
27	1	0	1	0	0	**2**	1	1	2	0	0	**4**	**6**
28	1	0	1	0	0	**2**	1	0	2	0	0	**3**	**5**
29	2	0	1	0	0	**3**	2	3	1	0	0	**6**	**9**
30	2	0	1	0	0	**3**	2	0	1	0	0	**3**	**6**
31	1	0	1	0	0	**2**	1	2	1	0	0	**4**	**6**
32	1	0	1	0	0	**2**	1	2	2	0	0	**5**	**7**
33	2	0	1	0	0	**3**	1	2	2	0	0	**5**	**8**
34	1	1	1	0	0	**3**	1	2	1	0	0	**4**	**7**
35	1	3	1	0	0	**5**	1	1	1	0	0	**3**	**8**
36	2	0	0	0	0	**2**	2	2	2	0	0	**6**	**8**
37	1	0	1	0	0	**2**	2	2	3	0	0	**7**	**9**
38	1	0	1	0	0	**2**	1	2	2	0	0	**5**	**7**
39	2	0	1	0	0	**3**	1	2	2	0	0	**5**	**8**
40	2	0	1	0	0	**3**	1	2	2	0	0	**5**	**8**
41	1	0	1	0	0	**2**	2	1	1	0	0	**4**	**6**
42	2	1	1	0	0	**4**	2	1	2	0	0	**5**	**9**
43	2	0	1	0	0	**3**	1	1	2	0	0	**4**	**7**
44	2	2	0	0	0	**4**	2	2	2	0	0	**6**	**10**
45	2	1	1	0	0	**4**	2	2	2	0	0	**6**	**10**
46	2	2	0	0	0	**4**	2	1	2	0	0	**5**	**9**
47	2	0	1	0	0	**3**	1	2	1	0	0	**4**	**7**
48	2	0	1	0	0	**3**	1	1	2	0	0	**4**	**7**
49	1	0	1	0	0	**2**	1	2	2	0	0	**5**	**7**
50	2	2	1	0	0	**5**	1	2	2	0	0	**5**	**10**

Supplementary Figure S1. Neutralization score. 10 types of group 1, group 2 including post-vaccine sera and vaccine strains (H1N1 (A/Puerto Rico/8/1934, A/California/04/2009, A/Guangdong-Maonan SWL1536/2019, A/Victoria/1/2020), H5N1 (A/Vietnam/1194/2004), H3N2 (A/X-31, A/Guizhou/54/1989, A/Hong Kong/2671/2019, A/Darwin/9/2021), and H7N9 (A/Anhui/1/2013)). Neutralizing antibody titers were measured. To quantify serum neutralizing titers, a score was assigned based on the neutralizing antibody titer of each virus strain (score 1 for titers between 21-80, score 2 for titers between 81-640, score 3 for titers between 641-1280, and score 4 for titers >1280). Score 1 is indicated in green, score 2 in yellow, score 3 in orange, and score 4 in red; the sum of scores for Group 1, Group 2, and Group 1+2 virus strains is indicated on the right.

ID	Group1 score						Group2 score						G1+2
	Vic	GM	Cal04	PR8	H5	**Total**	Dar	HK	GZ	X31	H7	**Total**	**Total**
1	2	2	0	0	2	**6**	0	2	2	1	0	**5**	**11**
2	2	2	0	0	1	**5**	0	2	2	2	0	**6**	**11**
3	2	2	0	0	2	**6**	0	2	2	2	1	**7**	**13**
4	1	3	0	0	1	**5**	0	3	1	2	0	**6**	**11**
5	2	3	1	1	2	**9**	2	4	2	2	2	**12**	**21**
6	2	1	0	0	1	**4**	0	4	2	2	1	**9**	**13**
7	1	2	0	0	1	**4**	0	3	2	2	0	**7**	**11**
8	1	2	0	0	2	**5**	0	3	2	2	4	**11**	**16**
9	2	1	1	0	2	**6**	0	3	2	2	0	**7**	**13**
10	2	3	0	0	4	**9**	4	4	3	2	0	**13**	**22**
11	0	0	0	0	0	**0**	0	2	1	0	1	**4**	**4**
12	1	2	0	0	2	**5**	0	3	1	2	0	**6**	**11**
13	1	3	0	0	2	**6**	0	2	1	3	1	**7**	**13**
14	1	2	0	0	2	**5**	2	2	2	3	1	**10**	**15**
15	3	3	0	0	2	**8**	0	4	2	4	0	**10**	**18**
16	2	1	0	0	1	**4**	0	2	1	2	0	**5**	**9**
17	2	3	1	0	2	**8**	1	3	1	2	0	**7**	**15**
18	2	2	0	0	2	**6**	0	3	3	2	0	**8**	**14**
19	4	4	1	0	3	**12**	0	4	3	3	0	**10**	**22**
20	1	1	0	0	0	**2**	0	2	2	1	2	**7**	**9**
21	2	3	0	0	2	**7**	0	3	2	3	0	**8**	**15**
22	2	4	0	2	3	**11**	0	2	2	2	0	**6**	**17**
23	2	2	0	1	0	**5**	1	2	2	2	0	**7**	**12**
24	2	2	0	0	2	**6**	0	3	2	3	0	**8**	**14**
25	3	2	0	0	3	**8**	0	2	2	2	1	**7**	**15**
26	1	2	0	0	1	**4**	0	1	2	2	0	**5**	**9**
27	2	1	0	0	1	**4**	4	2	2	2	0	**10**	**14**
28	1	2	0	0	2	**5**	4	2	2	2	0	**10**	**15**
29	3	1	1	0	2	**7**	4	2	2	3	0	**11**	**18**
30	2	2	1	0	2	**7**	0	4	3	2	0	**9**	**16**
31	2	2	0	0	1	**5**	4	2	2	3	0	**11**	**16**
32	1	2	0	0	2	**5**	4	4	3	2	0	**13**	**18**
33	0	2	0	0	2	**4**	0	2	2	3	0	**7**	**11**
34	1	1	0	0	1	**3**	0	2	2	3	2	**9**	**12**
35	2	2	0	0	1	**5**	0	1	1	3	0	**5**	**10**
36	2	4	1	0	3	**10**	0	4	2	2	0	**8**	**18**
37	1	2	0	0	3	**6**	1	4	3	2	0	**10**	**16**
38	0	2	0	0	2	**4**	0	3	2	2	0	**7**	**11**
39	1	2	0	0	1	**4**	0	4	2	2	0	**8**	**12**
40	0	3	0	1	3	**7**	0	4	2	3	0	**9**	**16**
41	0	1	0	0	1	**2**	0	2	2	3	1	**8**	**10**
42	1	2	0	0	2	**5**	0	2	1	1	2	**6**	**11**
43	0	2	1	0	2	**5**	0	2	2	2	0	**6**	**11**
44	1	2	0	0	1	**4**	0	4	2	1	0	**7**	**11**
45	1	2	0	0	1	**4**	0	4	2	2	0	**8**	**12**
46	1	3	0	0	2	**6**	1	3	3	2	2	**11**	**17**
47	1	3	0	0	3	**7**	0	2	1	1	0	**4**	**11**
48	2	2	0	0	2	**6**	0	2	2	1	0	**5**	**11**
49	1	2	0	0	2	**5**	0	2	2	2	0	**6**	**11**
50	2	3	0	0	2	**7**	0	3	2	1	0	**6**	**13**

Supplementary Figure S2. ADCC activation score. 10 types of group 1, group 2 including post-vaccine sera and vaccine strains (H1N1 (A/Puerto Rico/8/1934, A/California/04/2009, A/Guangdong-Maonan SWL1536/2019, A/Victoria/1/2020), H5N1 (A/Vi-etnam/1194/2004), H3N2 (A/X-31, A/Guizhou/54/1989, A/Hong Kong/2671/2019, A/Darwin/9/2021) and H7N9 (A/Anhui/1/2013)). ADCC activity was measured using ADCC activity, and scores were assigned based on a scale of 1 for titers between 11-100 ADCC activity, 2 for titers between 101-500, 3 for titers between 501-1000, and 4 for titers >1000. Score 1 is indicated in green, score 2 in yellow, score 3 in orange, and score 4 in red; the sum of scores for Group 1, Group 2, and Group 1+2 virus strains is indicated on the right.

ID	Group1 score						Group2 score						G1+2
	Vic	GM	Cal04	PR8	H5	**Total**	Dar	HK	GZ	X31	H7	**Total**	**Total**
1	0	2	0	0	2	**4**	0	1	1	1	0	**3**	**7**
2	0	1	0	0	0	**1**	2	1	1	0	0	**4**	**5**
3	0	1	0	0	1	**2**	1	1	1	1	0	**4**	**6**
4	0	2	0	0	0	**2**	1	2	0	0	0	**3**	**5**
5	0	2	0	0	2	**4**	1	3	1	2	0	**7**	**11**
6	0	0	0	0	0	**0**	1	1	2	0	0	**4**	**4**
7	0	1	0	0	1	**2**	0	2	1	0	0	**3**	**5**
8	0	1	0	0	1	**2**	0	2	1	1	1	**5**	**7**
9	0	0	0	0	1	**1**	1	1	1	0	1	**4**	**5**
10	0	2	0	0	3	**5**	0	0	2	0	0	**2**	**7**
11	0	0	0	0	0	**0**	0	0	1	0	1	**2**	**2**
12	0	2	0	0	2	**4**	0	2	0	1	0	**3**	**7**
13	0	2	0	1	2	**5**	0	1	0	1	1	**3**	**8**
14	0	1	0	0	2	**3**	2	2	1	0	0	**5**	**8**
15	0	2	0	0	2	**4**	0	2	1	1	0	**4**	**8**
16	0	1	0	0	0	**1**	0	1	0	0	0	**1**	**2**
17	0	1	0	0	2	**3**	0	2	0	1	0	**3**	**6**
18	0	1	0	0	2	**3**	0	1	1	0	0	**2**	**5**
19	0	2	0	0	2	**4**	0	2	1	0	0	**3**	**7**
20	0	0	0	0	0	**0**	0	1	0	0	0	**1**	**1**
21	0	2	1	0	1	**4**	0	2	1	0	0	**3**	**7**
22	1	2	1	2	3	**9**	1	2	1	1	1	**6**	**15**
23	0	2	0	0	2	**4**	1	1	1	1	1	**5**	**9**
24	0	1	0	0	1	**2**	0	1	1	0	0	**2**	**4**
25	0	1	2	0	1	**4**	0	1	2	1	0	**4**	**8**
26	0	1	0	0	1	**2**	1	1	1	1	0	**4**	**6**
27	0	0	0	0	0	**0**	1	0	2	0	0	**3**	**3**
28	0	1	0	0	2	**3**	1	1	1	0	1	**4**	**7**
29	0	0	0	0	0	**0**	0	0	1	1	0	**2**	**2**
30	0	1	4	0	0	**5**	1	0	1	1	0	**3**	**8**
31	0	0	0	0	0	**0**	0	1	0	1	0	**2**	**2**
32	0	2	0	1	0	**3**	0	2	1	1	0	**4**	**7**
33	0	1	0	1	2	**4**	1	1	1	1	1	**5**	**9**
34	0	1	0	0	1	**2**	0	1	1	1	0	**3**	**5**
35	0	1	0	0	1	**2**	0	1	1	1	0	**3**	**5**
36	0	4	0	0	1	**5**	1	0	1	0	1	**3**	**8**
37	0	1	0	0	3	**4**	0	2	3	1	1	**7**	**11**
38	0	1	0	0	2	**3**	0	2	1	0	0	**3**	**6**
39	0	1	0	0	0	**1**	0	0	2	0	0	**2**	**3**
40	0	2	4	0	1	**7**	2	0	2	4	0	**8**	**15**
41	0	1	0	0	1	**2**	0	0	2	1	1	**4**	**6**
42	0	1	0	0	2	**3**	0	1	1	0	1	**3**	**6**
43	0	2	0	0	1	**3**	0	1	1	0	0	**2**	**5**
44	1	0	0	2	0	**3**	1	0	1	0	0	**2**	**5**
45	0	0	4	0	0	**4**	0	0	2	1	0	**3**	**7**
46	0	3	2	0	2	**7**	0	0	2	1	1	**4**	**11**
47	0	1	2	0	1	**4**	0	0	1	1	0	**2**	**6**
48	0	1	4	0	2	**7**	0	1	0	0	0	**1**	**8**
49	0	1	2	0	1	**4**	1	1	1	0	0	**3**	**7**
50	0	1	0	0	1	**2**	0	0	1	0	0	**1**	**3**

Supplementary Figure S3. ADCP activation score. 10 types of group 1, group 2 including post-vaccine sera and vaccine strains (H1N1 (A/Puerto Rico/8/1934, A/California/04/2009, A/Guangdong-Maonan SWL1536/2019, A/Victoria/1/2020), H5N1 (A/Vietnam/1194/2004), H3N2 (A/X-31, A/Guizhou/54/1989, A/Hong Kong/2671/2019, A/Darwin/9/2021), and H7N9 (A/Anhui/1/2013)). ADCP activity was measured using ADCP activity, and scores were assigned based on a score of 1 for titers between 11-100 ADCP activity, 2 for titers between 101-500, score 3 for titers between 501-1000, and 4 for titers >1000. Score 1 is indicated in green, score 2 in yellow, score 3 in orange, and score 4 in red; the sum of scores for Group 1, Group 2, and Group 1+2 virus strains is indicated on the right.

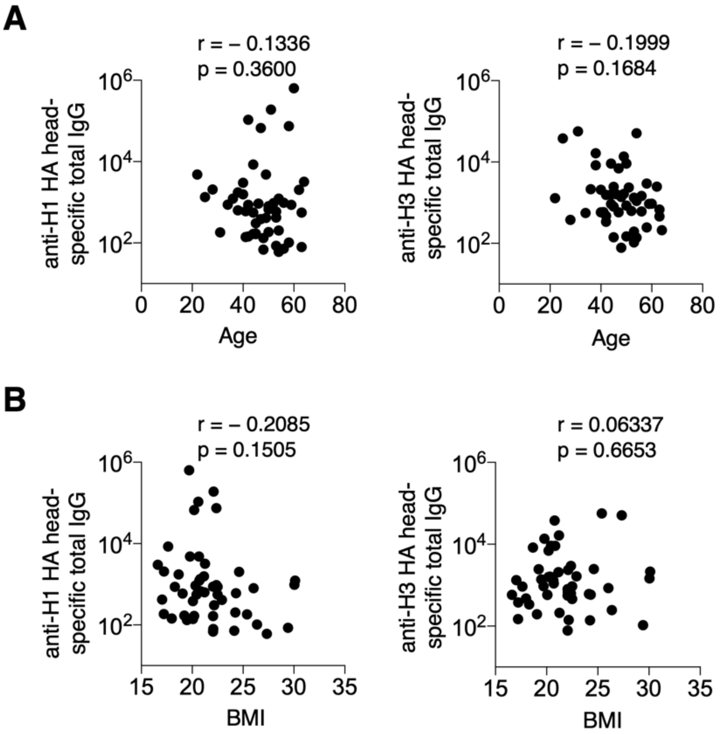

Supplementary Figure S4. Relationship between H1, H3 HA head-specific total IgG in serum, and Age or BMI.(A) Correlation between H1, H3 HA head-specific total IgG and Age. (B) Correlation between H1, H3 HA head-specific total IgG and BMI. Data were analyzed using Spearman's rank correlation coefficients. Lines indicate the correlations determined by linear regression analysis (n = 50).
